# The serological IgG and neutralizing antibody of SARS-CoV-2 omicron variant reinfection in Jiangsu Province, China

**DOI:** 10.3389/fpubh.2024.1364048

**Published:** 2024-05-30

**Authors:** Jinjin Chu, Qigang Dai, Chen Dong, Xiaoxiao Kong, Hua Tian, Chuchu Li, Jiefu Peng, Ke Xu, Hao Ju, Changjun Bao, Jianli Hu, Liguo Zhu

**Affiliations:** ^1^Department of Acute Infectious Disease Control and Prevention, Jiangsu Provincial Center for Disease Control and Prevention, Nanjing, China; ^2^National Health Commission (NHC) Key Laboratory of Enteric Pathogenic Microbiology, Jiangsu Provincial Center for Disease Control and Prevention, Nanjing, China; ^3^Jiangsu Province Engineering Research Center of Health Emergency, Nanjing, China; ^4^Jiangsu Key Lab of Cancer Biomarkers, Prevention and Treatment, Jiangsu Collaborative Innovation Center for Cancer Medicine, Nanjing Medical University, Nanjing, China

**Keywords:** SARS-CoV-2, omicron variant, reinfection, immunity, IgG, Nab, public health

## Abstract

**Background:**

It is important to figure out the immunity of Severe Acute Respiratory Syndrome Coronavirus 2 (SARS-CoV-2) reinfection to understand the response of humans to viruses. A serological survey for previously infected populations in Jiangsu Province was conducted to compare the antibody level of SARS-CoV-2 in reinfection by Omicron or not.

**Methods:**

Reinfection with SARS-CoV-2 was defined as an individual being infected again after 90 days of the initial infection. Telephone surveys and face-to-face interviews were implemented to collect information. Experimental and control serum samples were collected from age-sex-matched reinfected and non-reinfected cases, respectively. IgG anti-S and neutralizing antibodies (Nab) concentrations were detected by the Magnetism Particulate Immunochemistry Luminescence Method (MCLIA). Antibody titers were log(2)-transformed and analyzed by a two-tailed Mann–Whitney *U* test. Subgroup analysis was conducted to explore the relationship between the strain type of primary infection, SARS-Cov-2 vaccination status, and antibody levels. Multivariate linear regression models were used to identify associations between reinfection with IgG and Nab levels.

**Results:**

Six hundred thirty-one individuals were enrolled in this study, including 327 reinfected cases and 304 non-reinfected cases. The reinfection group had higher IgG (5.65 AU/mL vs. 5.22 AU/mL) and Nab (8.02 AU/mL vs. 7.25 AU/mL) levels compared to the non-reinfection group (*p* < 0.001). Particularly, individuals who had received SARS-CoV-2 vaccination or were initially infected with the Wild type and Delta variant showed a significant increase in antibody levels after reinfection. After adjusting demographic variables, vaccination status and the type of primary infection together, IgG and Nab levels in the reinfected group increased by log(2)-transformed 0.71 and 0.64 units, respectively (*p* < 0.001). This revealed that reinfection is an important factor that affects IgG and Nab levels in the population.

**Conclusion:**

Reinfection with Omicron in individuals previously infected with SARS-CoV-2 enhances IgG and Nab immune responses.

## 1 Introduction

An outbreak of coronavirus disease 2019 (COVID-19) caused by severe acute respiratory syndrome coronavirus 2 (SARS-CoV-2) emerged at the end of 2019 (1). Subsequently, SARS-CoV-2 rapidly triggered pandemics both domestically and internationally. Up to December 31, 2022, over 720 million confirmed COVID-19 cases and 6.7 million deaths were reported globally (2). China maintained a relatively low prevalence rate from 2019 to 2022. However, a new outbreak, dominated by the Omicron variant, accelerated the peak of infection in late 2022. Notably, more than 80% of the population was infected with the SARS-CoV-2 Omicron variant during the epidemic period (3). Consequently, the question of whether the immune response of a primary infected person can prevent re-infection has attracted the attention of the whole society.

Previous studies have indicated that SARS-CoV-2 antibody-positivity acquired from infection can be maintained for at least 6 months, and reinfection with SARS-CoV-2 was infrequent (4, 5). With virus mutation and antibody attenuation, there has been a significant increase in SARS-CoV-2 reinfection cases was observed nationwide in December 2022. Reinfections gradually became a common concern. Unfortunately, to date, there is limited knowledge about the serologic characteristics of reinfection with SARS-CoV-2 in individuals who were originally infected. The lack of data regarding SARS-CoV-2 reinfection poses a challenge for promptly adjusting our response and vaccination strategies, which is essential in effectively suppressing local outbreaks of the epidemic in the future. Therefore, there was an urgent need to conduct a serological investigation to figure out the serological characteristics among these people reinfected in a pandemic.

Serological testing can be of great help in the prevention and control of COVID-19 outbreaks, including diagnosis of infection, determination of the seroprevalence of SARS-CoV-2 infection in a population, and assessment of immune response induced by vaccination or SARS-CoV-2 infection (6, 7). In this study, both epidemiological and serological investigations were conducted simultaneously. On the one hand, concise information on cases was obtained by a telephone survey and further interview was conducted to collect detailed information on eligible subjects. First, a telephone survey provided concise information on cases, and detailed information was then collected through interviews with eligible subjects. Additionally, population-based surveys of antibody levels were carried out to analyze the immune response triggered by the Omicron variant. We hope that this study will provide insight into how individual antibody levels change during a pandemic, and offer suggestions for actively combating the virus.

## 2 Methods

### 2.1 Study design and participants

**Figure 1:**
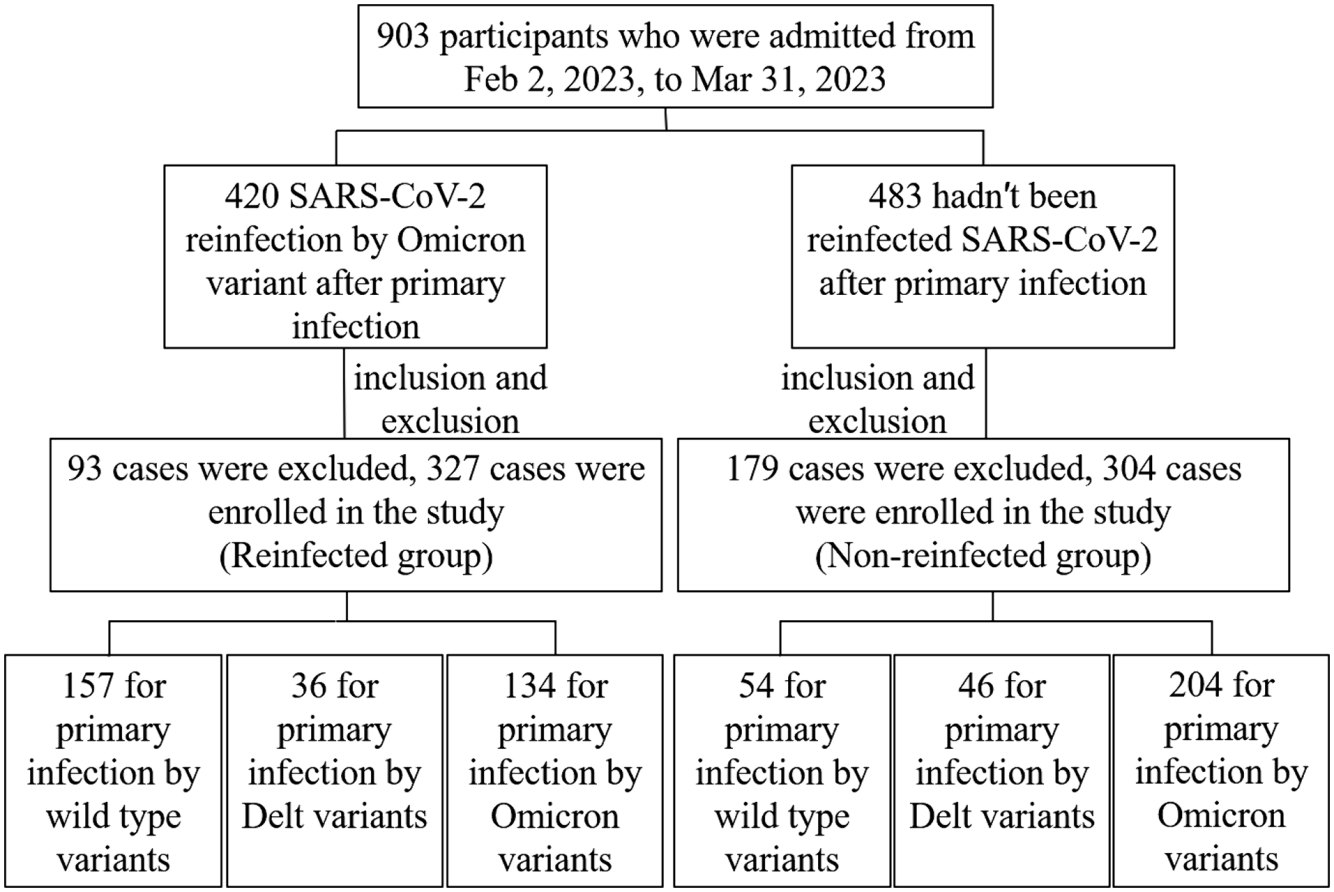
Flow chart of enrollment of study subjects.

The SARS-CoV-2 reinfection investigation was implemented in Jiangsu province, aiming to probable or confirmed SARS-CoV-2 cases reported by China Information System for Disease Control and Prevention from 22 January 2020 to 1 December 2022. A telephone survey was used to figure out if a reinfection had occurred in December 2022. If they self-reported a reinfection, the face-to-face interviews will be implemented by uniformly trained staff to collect detailed information, serum samples and antibody detection. Some of those who were not reinfected will be recruited into the control group according to age-sex-matched reinfected (Supplementary Figure 1). A total of 631 cases eventually were recruited in this study (Figure 1). A 5 mL peripheral blood sample was collected from each participant by anticoagulant blood vessel with EDTA. The serum was separated from the whole blood within 12 h after collection and stored at −80°C for further analysis. All the participants met the following criteria: (a) voluntarily participation in this study with verbal or signed informed consent; (b) the strain types of primary SARS-CoV-2 infection were confirmed by gene sequences; (c) vaccination information was documented clearly. Exclusion criteria for participants: (a) specimen hematolysis; (b) participants missing demographic information or serum samples.

SARS-CoV-2 infection was defined as a person who is positive for SARS-CoV-2 antigen or nucleic acid or possesses SARS-CoV-2 relevant symptoms and a history of epidemiology (8); SARS-CoV-2 reinfection was defined as the span period of infection occurring or two positive specimens on a patient collected at least 90 days according to U.S. CDC guidance (9). Serum samples were collected from those age-sex-matched controls without reinfection.

### 2.2 SARS-CoV-2 IgG and neutralizing antibodies test

Magnetism Particulate Immunochemistry Luminescence Method (MCLIA) was used to detect the IgG anti-S and neutralizing antibodies (Nab) in serum samples by an automated Axceed 260 analyzer. The titers of SARS-CoV-2-specific antibodies IgG were evaluated by the IgG kits named”Diagnostic Kit for Novel Coronavirus (2019-nCoV) IgG Antibody (Magnetic particle CLIA).” Surrogate virus neutralization test titers were identified *in vitro* using receptor- binding domain (RBD-NAbs) of wild-type virus utilizing the competitive chemiluminescence method, with accompanying immunoassay test kits. (1) SARS-CoV-2 IgG test: the test kit consisted of reagent 0 (Magnetic Particles-Anti-FITC Antibody), reagent 1 (SARS-CoV-2 recombinant antigen labeled by FITC), reagent 2 (Mouse Anti-human IgG monoclonal antibody labeled by alkaline phosphatase), and other necessary auxiliary reagents. Firstly, add reagent 0, reagent 1 and the sample into the reaction tube. If the sample contains SARS-CoV-2 IgG, it will form a complex with recombinant antigen in the above reagent and bind to magnetic particles at the same time, while the free components will be washed away. After adding Reagent 2 into the reaction tube, the alkaline phosphatase labeled antibody acted as a secondary antibody to bind to the IgG antibody in the sample and then formed an antibody complex. Finally, alkaline phosphatase catalyzed the substrate solution to emit light, and the relative light units (RLU) of each sample tube were determined. The RLU was positively correlated with the concentration of SARS-CoV-2 IgG in the sample (10). (2) Surrogate virus neutralization test: the competitive chemiluminescence method was adopted as a previous study described (11). The test kit included regent 0 (magnetic particle receptor angiotensin-converting enzyme (ACE2) Antigen), regent 1 (S protein RBD labeled by alkaline phosphatase), calibrator, and other necessary auxiliary reagents. Reagent 0, reagent 1, and serum samples were added to the reaction tubes together, and then the substrate solution was catalyzed by the alkaline phosphatase to emit light. If the sample contained NAb, it would compete with magnetic particle-labeled ACE2 antigen to bind S protein RBD. Finally, the concentration of SARS-CoV-2-NAb in each sample was quantified according to the relationship between the RLU and the concentration of SARS-CoV-2-NAb in the human serum sample.

### 2.3 Statistical analysis

IBM SPSS Statistics 24 and GraphPad Prism 8 were used together for statistical analysis. IBM SPSS was used to analyze the data and GraphPad Prism8 was mainly used to present the result by drawing diagrams. Antibody titers were log(2)-transformed before analysis. Continuous variables were described as the mean ± standard deviation ( $\bar{x}$ ± s) or median and interquartile range [M (Q1, Q3)]. Categorical variables were summarized as counts and percentages. The two-tailed Mann–Whitney U test analyzed continuous, non-parametric variables. Categorical variables were analyzed by *χ*^2^ test. Adjusting linear regression models were used to identify factors associated with Nab levels among all participants. Unadjusted and adjusted odds ratios were calculated. We adjusted models for demographic variables (age and sex) and serum antibody-related variables (vaccination status, interval from last vaccination and the type of primary infection). *p* < 0.05 were considered statistically significant.

## 3 Results

### 3.1 Study design and participants

From February 2, 2023, to March 31, 2023, 903 serum samples were collected from the whole 13 cities in Jiangsu Province. According to the standard of inclusion and exclusion, 631 samples were finally enrolled in the study, including 327 reinfected individuals in the case group and 304 primary infected individuals in the control group (Figure 1). Participants consisted of 288 males and 343 females, ranging from 1 to 91 years old. Immunization information was also documented. More than 90% of participants had been vaccinated with the COVID-19 vaccine, but most of them had been vaccinated more than a year since their last vaccination. The demographic and characteristics of participants are shown in Table 1.

**Table 1:** Demographics and characteristics of all the participants.

Variables	Total	Non-reinfection	Reinfection	*χ*^2^/*Z*	p
Gender				0.002	0.968
Male	288 (45.64)	139 (45.72)	149 (45.57)		
Female	343 (54.36)	165 (54.28)	178 (54.43)		
Age (years)				0.639	0.726
0~	58 (9.19)	30 (9.87)	28 (8.56)		
18~	484 (76.70)	229 (75.33)	255 (77.98)		
60~	89 (14.10)	45 (14.80)	44 (13.46)		
SARS-Cov-2 vaccination status				16.103	0.001
Unvaccinated	67 (10.62)	29 (9.54)	38 (11.62)		
Incomplete	73 (11.57)	20 (6.58)	53 (16.21)		
complete	197 (31.22)	101 (33.22)	96 (29.36)		
Booster	294 (46.59)	154 (50.66)	140 (12.23)		
Interval from last vaccination				1.174	0.556
Unvaccinated	67 (10.62)	29 (8.87)	38 (12.50)		
≤12 months	182 (28.84)	85 (25.99)	96 (31.58)		
>12 months	382 (60.54)	190 (58.10)	192 (63.16)		
The type of Primary infection				65.245	<0.001
Wild type variants	211 (33.44)	54 (17.76)	157 (48.01)		
Delta variants	82 (13.00)	46 (15.13)	36 (11.01)		
Omicron variants	338 (53.57)	204 (67.11)	134 (40.98)		
The levels of IgG anti-S [log2 AU/mL,**M(Q1, Q3)]**	5.45 (4.39, 6.13)	5.22 (4.02, 5.93)	5.67 (4.71, 6.28)	−4.904	<0.001
The levels of Nab anti-S [log2 AU/mL, M (**Q1, Q3)]**	7.65 (6.21, 8.64)	7.25 (5.83, 8.21)	8.01 (6.75, 9.07)	−5.461	<0.001

### 3.2 Clinical symptoms

**Figure 2:**
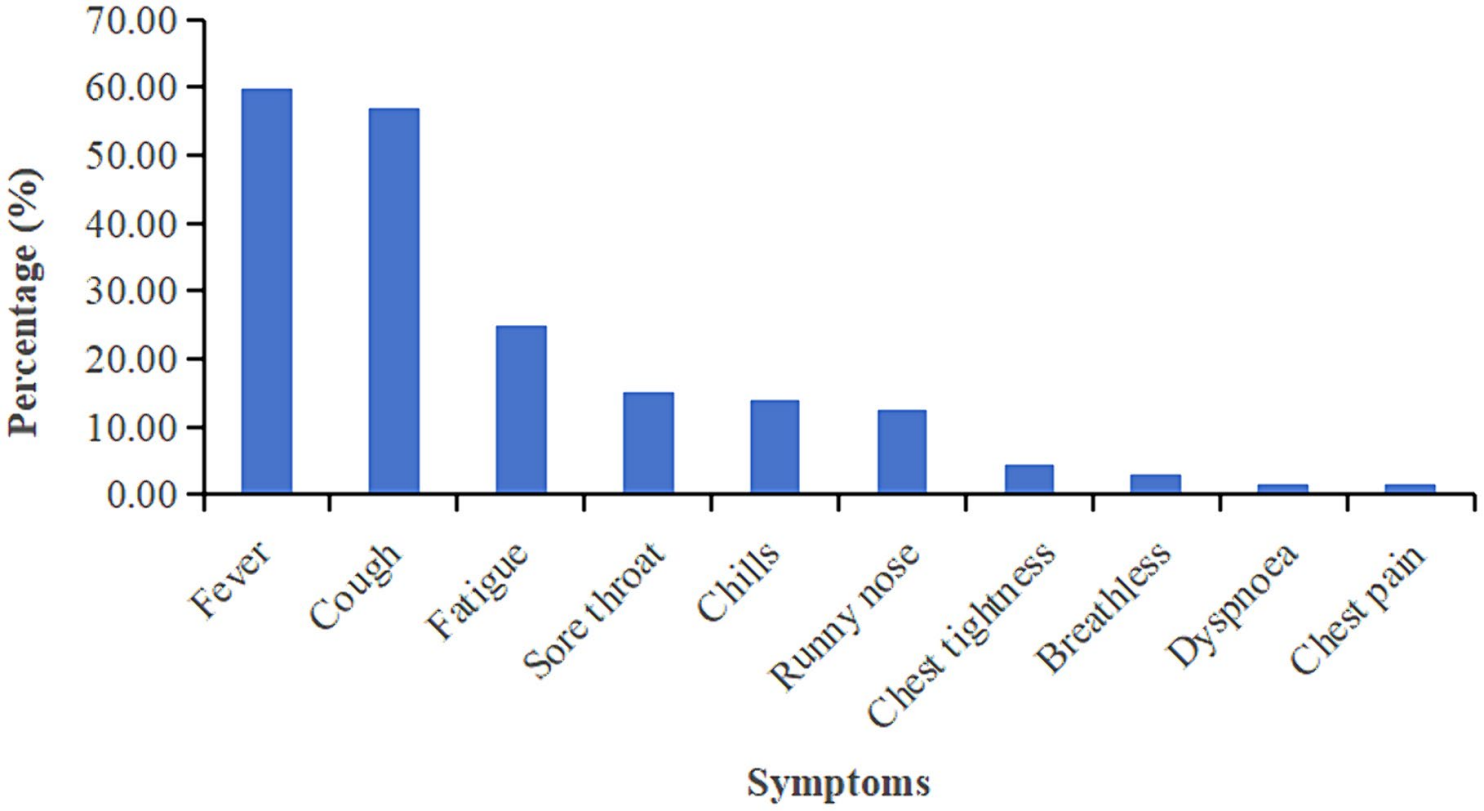
Percentage of patients with clinical symptoms to Omicron.

In this study, detailed symptom information for 327 reinfected cases was available. 96.94% (317/327) reinfected patients developed symptoms related to Omicron. The most common symptom is fever during the second infection (59.94%), followed by cough (56.88%) and fatigue (24.77%). The prevalence of detailed symptoms is presented in Figure 2. We further to identify relationship between symptoms and antibody levels by liner regression model the results showed that Symptomatic patients showed higher IgG level compared to asymptomatic infection, no significant differences between symptoms and Nab level were observed (Supplementary Table S1).

### 3.3 Antibody levels between reinfection and non-reinfection groups

**Figure 3:**
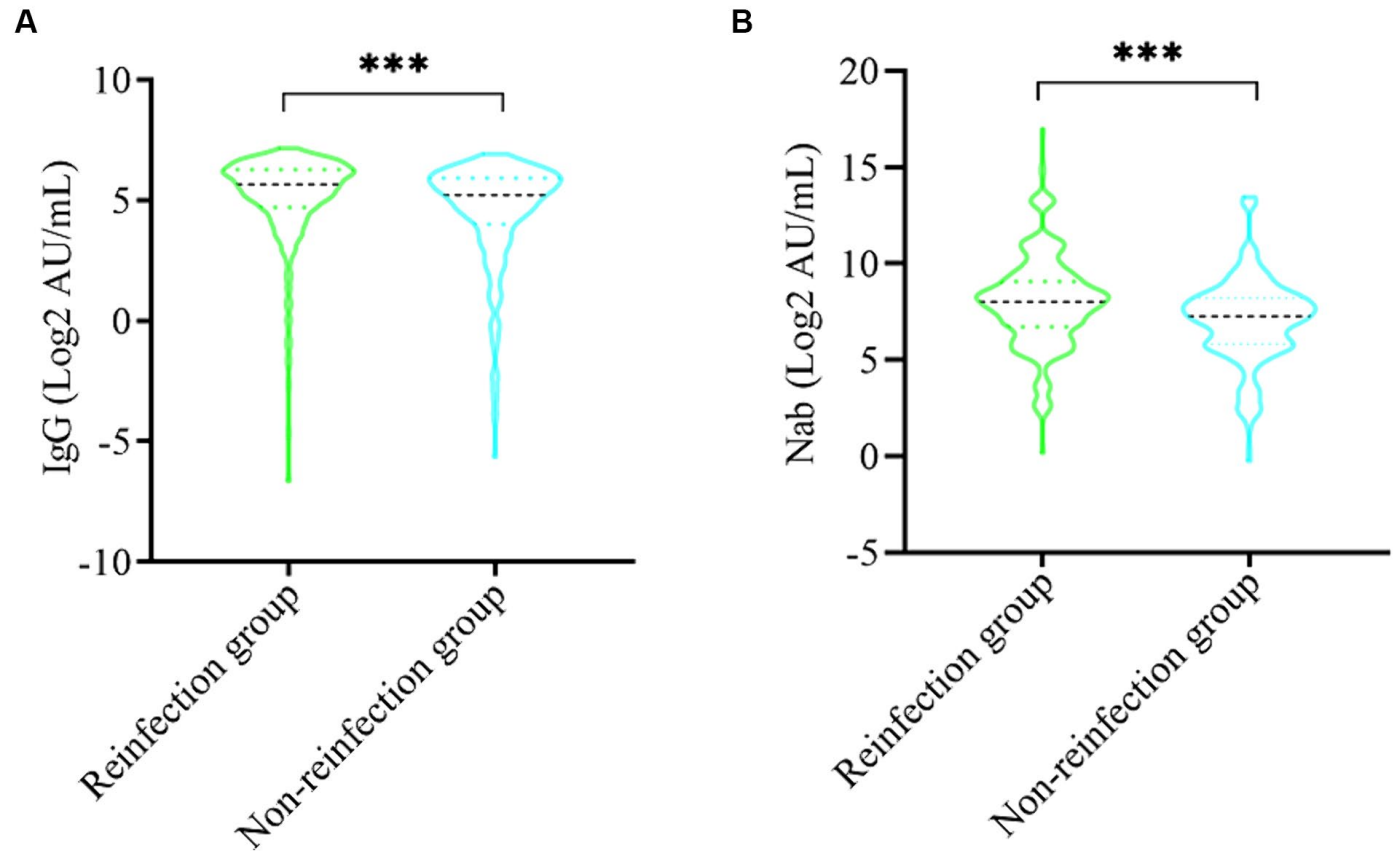
Comparison of SARS-CoV-2 serum antibodies between the reinfection group and non-reinfection. **(A)** Comparison of SARS-CoV-2 serum-IgG between the two groups. **(B)** Comparison of SARS-CoV-2 Nab between the two groups (^***^indicates *p* < 0.001).

To better demonstrate the antibody level of participants among different groups, we draw violins to show antibody levels of IgG and neutralizing antibodies (Nab), respectively. The antibody levels of IgG anti-S (log2 AU/mL) and Nab (log2 AU/mL) were much higher in the reinfected group than non-reinfected group (*p* < 0.05). The average concentrations (log2 AU/mL) of IgG and Nab of the reinfected group were 8.01 (6.75, 9.07) AU/mL and 7.25 (5.83, 8.21) AU/mL, respectively, while were 5.67 (4.71, 6.28) AU/mL and 5.22 (4.02, 5.93) AU/mL for a non-reinfection group (Figure 3).

### 3.4 Antibody levels between reinfection and non-reinfection groups according to the types of primary SARS-CoV-2 infection

**Figure 4:**
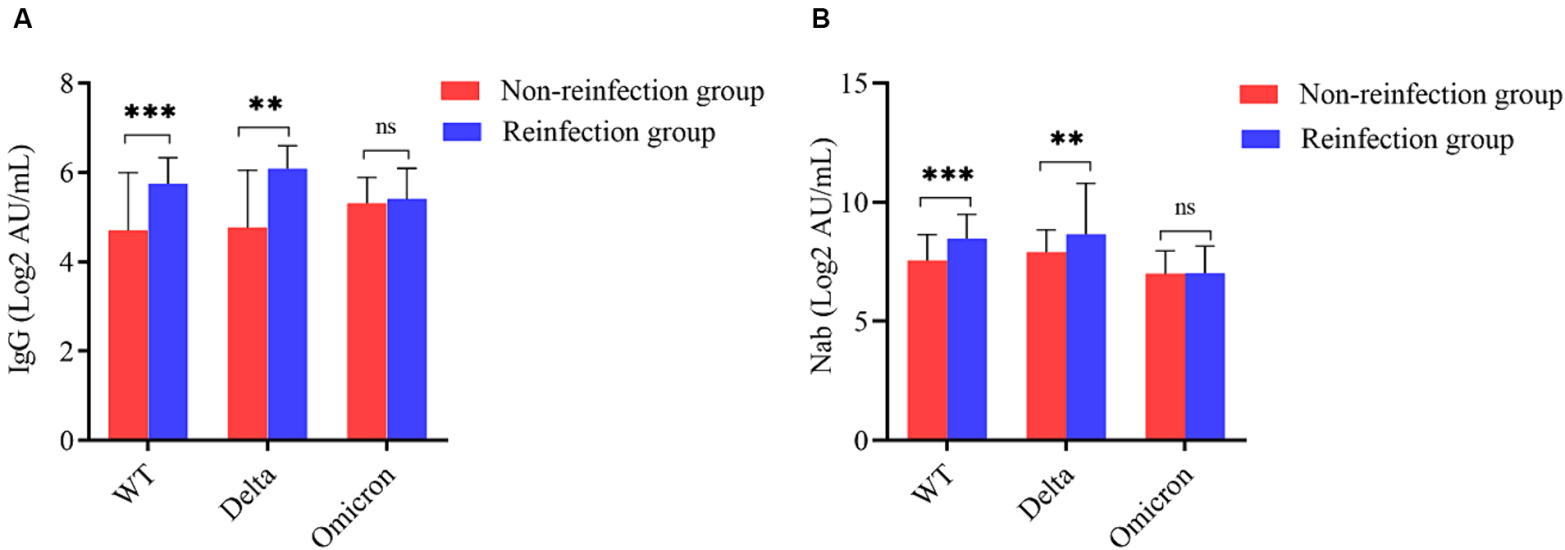
Subgroup analysis based on the type of primary SARS-CoV-2 infection. **(A)** Comparison of SARS-CoV-2 serum-IgG between the two groups according to primary infection strain. **(B)** Comparison of SARS-CoV-2 Nab between the two groups according to primary infection strain (^**^indicates *p* < 0.05, ^***^indicates *p* < 0.001, ^ns^ indicates *p* > 0.05).

After comparing the antibody level between the reinfected group and the non-reinfected group, we sought to further explore whether SARS-CoV-2 reinfection by the Omicron variant will elicit higher IgG and Nab response. Participants were classified into three subgroups according to the types of primary SARS-CoV-2 infection: the wild-type strain, the Delta variants, and the Omicron variant. When categorized by the wild-type strain and the Delta variants, both average concentrations of IgG and Nab exhibited significantly higher in the reinfected group than in the non-reinfected group (*p* < 0.05). The reinfected cases with primary Delta strain infection showed the highest antibody levels, the average concentrations of IgG and Nab were 6.08 (4.37, 6.60) AU/mL and 8.65 (7.98, 10.79) AU/mL, respectively. However, there were no statistically significant between the reinfected group and the non-reinfected group when categorized by the Omicron variant (*p* > 0.05) (Figure 4).

### 3.5 Antibody levels between reinfection and non-reinfection groups according to SARS-Cov-2 vaccination status

**Figure 5:**
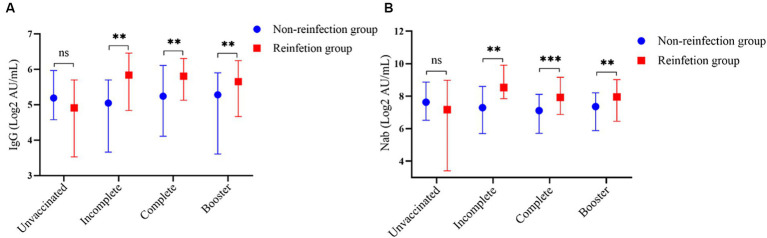
Subgroup analysis based on the SARS-CoV-2 vaccination status. **(A)** Comparison of SARS-CoV-2 serum-IgG between the two groups according to the SARS-Cov-2 vaccination status. **(B)** Comparison of SARS-CoV-2 Nab between the two groups according to the SARS-CoV-2 vaccination status (^**^indicates *p* < 0.05, ^***^indicates *p* < 0.001, ^ns^ indicates *p* > 0.05).

Participants were classified into four subgroups according to SARS-Cov-2 vaccination status: Unvaccinated, Incomplete, Complete, and Booster. Among all participants, the unvaccinated individuals in the reinfection group showed the lowest levels of antibodies, the average concentrations of IgG and Nab were 4.91 (3.53, 5.70) AU/mL and 7.17 (3.41, 8.97) AU/mL, respectively. Except for the unvaccinated individual’s subgroup, antibody levels in the reinfected group were higher than in the non-reinfection group (*p* < 0.05) (Figure 5).

### 3.6 Linear regression analysis of the factors influencing IgG and nab levels

Multivariate linear regression analysis was conducted to quantify the contributions of SARS-CoV-2 reinfection by the Omicron variant to IgG and Nab titers in all the participants. For the sake of controlling confounding bias, demographic variables including age and sex were first adjusted. Compared with the non-reinfected group, the IgG and Nab levels of the reinfected group increased by log(2)-transformed 0.66 and 0.94 units, respectively (Supplementary Table S2). After adjusting vaccination status and the type of primary infection, the results showed that the IgG and Nab levels increased by log(2)-transformed 0.70 and 0.64 units in the reinfected group compared to the non-reinfected group, respectively (Supplementary Table S3). Finally, reinfection was found to be a factor influencing IgG and Nab levels in all participants after adjusting demographic variables, vaccination status, and the type of primary infection together, the IgG and Nab levels increased by log(2)-transformed 0.71 and 0.64 units in the reinfected group compared to the non-reinfected group, respectively (Table 2).

**Table 2:** Adjusted multiple linear regression analysis of the relationship between SARS-CoV-2 reinfection and serum antibody levels.

Variables	Multiple Linear Regression Analysis (IgG)			*p*-value	Multiple Linear Regression Analysis (Nab)			*p*-value
	Unstandardized Coefficient		Standardized Coefficient		Unstandardized Coefficient		Standardized Coefficient	
	B	Standard error	β		B	Standard error	β	
Reinfection								
No	Reference				Reference			
Yes	0.71	0.17	0.18	<0.001	0.64	0.18	0.14	<0.001
Gender								
Male	Reference				Reference			
Female	−0.35	0.16	−0.09	0.03	−0.19	0.17	−0.04	0.264
Age (years)								
<18	Reference				Reference			
18~	0.05	0.28	0.01	0.87	0.47	0.31	0.09	0.125
60~	0.28	0.35	0.05	0.42	0.22	0.38	0.03	0.552
SARS-Cov-2 vaccination status								
Unvaccinated	Reference				Reference			
Incomplete	0.25	2.03	0.04	0.90	2.23	2.18	0.31	0.305
complete	0.02	2.02	0.01	0.99	1.99	2.17	0.40	0.361
Booster	−0.01	2.01	0.00	1.00	1.97	2.16	0.43	0.362
Interval from last vaccination								
Unvaccinated	Reference				Reference			
<12 months	−0.11	2.00	−0.02	0.96	−1.47	2.15	−0.29	0.494
>12 months	0.19	2.00	0.05	0.93	−1.37	2.15	−0.29	0.524
The type of Primary infection								
Wild type variants	Reference				Reference			
Delta variants	−0.05	0.27	−0.01	0.87	0.48	0.29	0.07	0.097
Omicron variants	0.27	0.20	0.07	0.16	−1.18	0.21	−0.26	<0.001

## 4 Discussion

The COVID-2019 pandemic has been going on for more than three years, raising concerns about whether mutations in the viral genome will result in the continued global emergence of the SARS-CoV-2 variant (12). The predominant strain of SARS-CoV-2 in China changed from the wild-type strain in 2020 to the Alpha and Delta variant in 2021, followed by the emergence of the Omicron variant in 2022. Currently, the Omicron variant and its sublines are the dominant strains. The Omicron variant was first identified in Zhenjiang, Jiangsu province, China on January 2, 2022, which subsequently became a dominant strain and caused a local outbreak. By the end of 2022, the Omicron variant had sparked a full-scale pandemic that severely impacted human health and life.

After the spreading of the Omicron variant, the number of infected individuals boomed in December 2022 (13), which was attributed to the waning of post-infection and post-vaccination immunity in the general population (14, 15). With the increasing number of SARS-CoV-2 infected cases and reinfections, there are growing concerns about the body’s immune response to the virus, and in particular the duration of protection against re-infection by Omicron-acquired immunity (16, 17). To gain insight into the serological characteristics following the pandemic, we conducted a population-based serological survey in Jiangsu Province.

IgG and Nab are the main antibodies protecting humans from SARS-CoV-2 infection.Previous studies have demonstrated that protective immunity could be assessed based on the correlation between serum-neutralizing antibody and IgG (18–20). High Nab concentrations showed attenuated viral RNA levels in both respiratory and gastrointestinal tracts, which led to rapid viral clearance and prevented the worsening of symptoms and hospitalization. Both IgG and Nab were detected for each subject by the surrogate virus neutralization test (MCLIA) in this study. The results suggested that the reinfection group produced higher antibody levels compared to the non-reinfection groups. This indicates reinfection may also stimulate the body’s immune response, reducing the viral load and infection disease severity. Clinicians are better able to judge the progression of COVID-19 patients based on monitoring antibody levels.

Additionally, a study conducted by Pilz S, et al. (5) revealed that immunological memory can be maintained for more than 6 months after infection. Based on this, we hypothesized that the likelihood is low for the reinfected population infected with SARS-CoV-2 Omicron in the short term. On the contrary, the non-infection group had a consistently higher risk of infection due to decreased immunity. However, SARS-CoV-2 has been continuously evolving in its viral genome, resulting in the emergence of new variants, the longevity of immunity post-reinfection against infection with new variants remained unknown. So, the importance of maintaining vigilant surveillance of emerging SARS-CoV-2 variants and exploring the cross-protective effects of antibody response post-reinfection.

Further comparisons were made between the reinfection group and non-reinfection group by dividing participants into three groups according to the strain types of primary infection. The reinfection groups exhibited significantly higher concentrations of IgG and Nab in both the wild-type and Delta variants. However, there was little or no increase in antibody levels among cases reinfected by Omicron. The results were consistent with the study conducted by Kim YI, et al. (20), whose animal model study among ferrets showed that reinfection with a heterologous strain induced higher IgG and Nab concentrations compared to primary infection. The results may be due to the short time interval between the second and first infections, as well as the fact that the serum antibody of the non-reinfection group had not yet fully declined. At the same time, we speculated that the human immune response to antigens will be more intensive when SARS-CoV-2 infected individuals are re-exposed to homologous antigens.

Although reinfection can occur for both vaccinated and unvaccinated individuals, this study found that vaccinated individuals had higher antibody levels than unvaccinated individuals. This result revealed that vaccination played an important role in eliciting the body’s immune response in individuals who were previously infected. A recent study reported that reinfection-acquired immunity boosted with vaccination will remain longer (21). Therefore, it is essential to expand SARS-CoV-2 vaccination for eligible persons, including those with previous infection, to reduce the risk of being reinfected.

The main limitation of this study is that it was a cross-sectional survey conducted after a pandemic. Antibodies titers were identified by ancestral RBD, which is different from Omicron RBD, and serum antibody levels in the population reinfected by Omicron were not continuously measured. Hence, we cannot accurately answer the question of how long the antibodies acquired through SARS-CoV-2 reinfection will last. Meanwhile, the threshold values for IgG, Nab and other serum antibodies to protect people against SARS-CoV-2 infection were unclear. Moreover, the results in this study cannot be generalized to broader populations owing to the convenient sampling and some information bias.

In a word, this study found that individuals reinfected with SARS-CoV-2 Omicron had higher IgG and Nab concentrations, indicating Omicron would be difficult to trigger a pandemic in a short time. Future research should focus on regular follow-up surveys and dynamic observation of changes in serum antibodies after reinfection. In addition, further experiments are also required to explore the cross-protective effect of Omicron-induced antibodies.

## Data availability statement

The individual case data in this article are the property of Acute Infectious Disease Control and Prevention Institute of JiangSu Provincial Centre For Disease Control And Prevention. If you want to access that data, please contact the corresponding authors for further information.

## Ethics statement

The studies involving humans were approved by Ethics Committee of Jiangsu Provincial Center for Disease Prevention and Control. The studies were conducted in accordance with the local legislation and institutional requirements. The participants provided their written informed consent to participate in this study.

## Author contributions

JC: Conceptualization, Data curation, Software, Writing – original draft, Writing – review & editing. QD: Data curation, Writing – review & editing. CD: Methodology, Writing – review & editing. XK: Methodology, Writing – review & editing. HT: Methodology, Writing – review & editing. CL: Methodology, Writing – review & editing. JP: Resources, Writing – review & editing. KX: Resources, Writing – review & editing. HJ: Writing – review & editing. CB: Writing – review & editing. JH: Writing – review & editing, Funding acquisition. LZ: Supervision, Validation, Writing – review & editing.
