# Supplementary material for: The serological IgG and neutralizing antibody of SARS-CoV-2 omicron variant reinfection in Jiangsu Province, China
Source: Front Public Health. 2024 May 30;12:1364048. doi: 10.3389/fpubh.2024.1364048 (PMC11169644; doi:10.3389/fpubh.2024.1364048)
Supplement: Supplementary file 1 [file Table_1.DOC]

Table S1 the probability of symptomatic infection associated with IgG and Nab levels

| Variables | Linear Regression Analysis (IgG) | | Standardized Coefficient | P-value | Linear Regression Analysis (Nab) | | Standardized Coefficient | P-value |
| --- | --- | --- | --- | --- | --- | --- | --- | --- |
|  | Unstandardized Coefficient | |  |  | Unstandardized Coefficient | |  |  |
|  | B | Standard error | β |  | B | Standard error | β |  |
| **Symptoms** |  |  |  | 0.046 |  |  |  | 0.263 |
| No | Reference |  |  |  | Reference |  |  |  |
| Yes | 1.159 | 0.578 | 0.111 |  | 0.832 | 0.742 | 0.062 |  |
