# Supplementary material for: The serological IgG and neutralizing antibody of SARS-CoV-2 omicron variant reinfection in Jiangsu Province, China
Source: Front Public Health. 2024 May 30;12:1364048. doi: 10.3389/fpubh.2024.1364048 (PMC11169644; doi:10.3389/fpubh.2024.1364048)
Supplement: Supplementary file 2 [file Table_2.DOC]

Table S2 Multiple linear regression analysis adjusting for demographic variables

| **Variables** | **Multiple Linear Regression Analysis (IgG)** | | | ***P***-value | **Multiple Linear Regression Analysis (Nab)** | | | ***P***-value |
| --- | --- | --- | --- | --- | --- | --- | --- | --- |
|  | **Unstandardized Coefficient** | | **Standardized Coefficient** |  | **Unstandardized Coefficient** | | **Standardized Coefficient** |  |
|  | ***B*** | **Standard error** | ***β*** |  | ***B*** | **Standard error** | ***β*** |  |
| **Reinfection** |  |  |  |  |  |  |  |  |
| No | Reference |  |  |  | Reference |  |  |  |
| Yes | 0.66 | 0.16 | 0.16 | <0.001 | 0.94 | 0.18 | 0.21 | <0.001 |
| **Gender** |  |  |  |  |  |  |  |  |
| Male | Reference |  |  |  | Reference |  |  |  |
| Female | -0.38 | 0.16 | -0.09 | 0.018 | -0.14 | 0.18 | -0.03 | 0.435 |
| **Age (years)** |  |  |  |  |  |  |  |  |
| **<18** | Reference |  |  |  | Reference |  |  |  |
| **18~** | -0.02 | 0.28 | -0.01 | 0.941 | 0.67 | 0.31 | 0.12 | 0.031 |
| **60~** | 0.14 | 0.34 | 0.02 | 0.676 | 0.78 | 0.38 | 0.12 | 0.040 |
