# Supplementary material for: The serological IgG and neutralizing antibody of SARS-CoV-2 omicron variant reinfection in Jiangsu Province, China
Source: Front Public Health. 2024 May 30;12:1364048. doi: 10.3389/fpubh.2024.1364048 (PMC11169644; doi:10.3389/fpubh.2024.1364048)
Supplement: Supplementary file 3 [file Table_3.DOC]

Table S3 Multiple linear regression analysis adjusting for relevant factors affecting SARS-CoV-2 antibody levels

| **Variables** | **Multiple Linear Regression Analysis (IgG)** | | | ***P***-value | **Multiple Linear Regression Analysis (Nab)** | | | ***P***-value |
| --- | --- | --- | --- | --- | --- | --- | --- | --- |
|  | **Unstandardized Coefficient** | | **Standardized Coefficient** |  | **Unstandardized Coefficient** | | **Standardized Coefficient** |  |
|  | ***B*** | **Standard error** | ***β*** |  | ***B*** | **Standard error** | ***β*** |  |
| **Reinfection** |  |  |  |  |  |  |  |  |
| No | Reference |  |  |  | Reference |  |  |  |
| Yes | 0.70 | 0.17 | 0.17 | <0.001 | 0.64 | 0.18 | 0.14 | <0.001 |
| **SARS-Cov-2 vaccination status** |  |  |  |  |  |  |  |  |
| Unvaccinated | Reference |  |  |  | Reference |  |  |  |
| Incomplete | -0.08 | 2.03 | -0.01 | 0.577 | 2.22 | 2.18 | 0.31 | 0.308 |
| complete | 0.16 | 2.02 | 0.04 | 0.017 | 1.92 | 2.17 | 0.39 | 0.377 |
| Booster | 0.16 | 2.01 | 0.04 | 0.008 | 1.96 | 2.16 | 0.43 | 0.366 |
| **Interval from last vaccination** |  |  |  |  |  |  |  |  |
| Unvaccinated | Reference |  |  |  | Reference |  |  |  |
| <12 months | 0.50 | 0.31 | 0.10 | 0.104 | -1.45 | 2.15 | -0.29 | 0.501 |
| >12 months | 0.59 | 0.27 | 0.14 | 0.031 | -1.34 | 2.15 | -0.29 | 0.534 |
| **The type of Primary infection** |  |  |  |  |  |  |  |  |
| Wild type variants | Reference |  |  |  | Reference |  |  |  |
| Delta variants | 0.26 | 0.27 | 0.04 | 0.331 | 0.42 | 0.29 | 0.06 | 0.152 |
| Omicron variants | -0.08 | 0.19 | -0.02 | 0.688 | -1.19 | 0.21 | -0.26 | <0.001 |
