# Supplementary figures and images for: The serological IgG and neutralizing antibody of SARS-CoV-2 omicron variant reinfection in Jiangsu Province, China
Source: Front Public Health. 2024 May 30;12:1364048. doi: 10.3389/fpubh.2024.1364048 (PMC11169644; doi:10.3389/fpubh.2024.1364048)

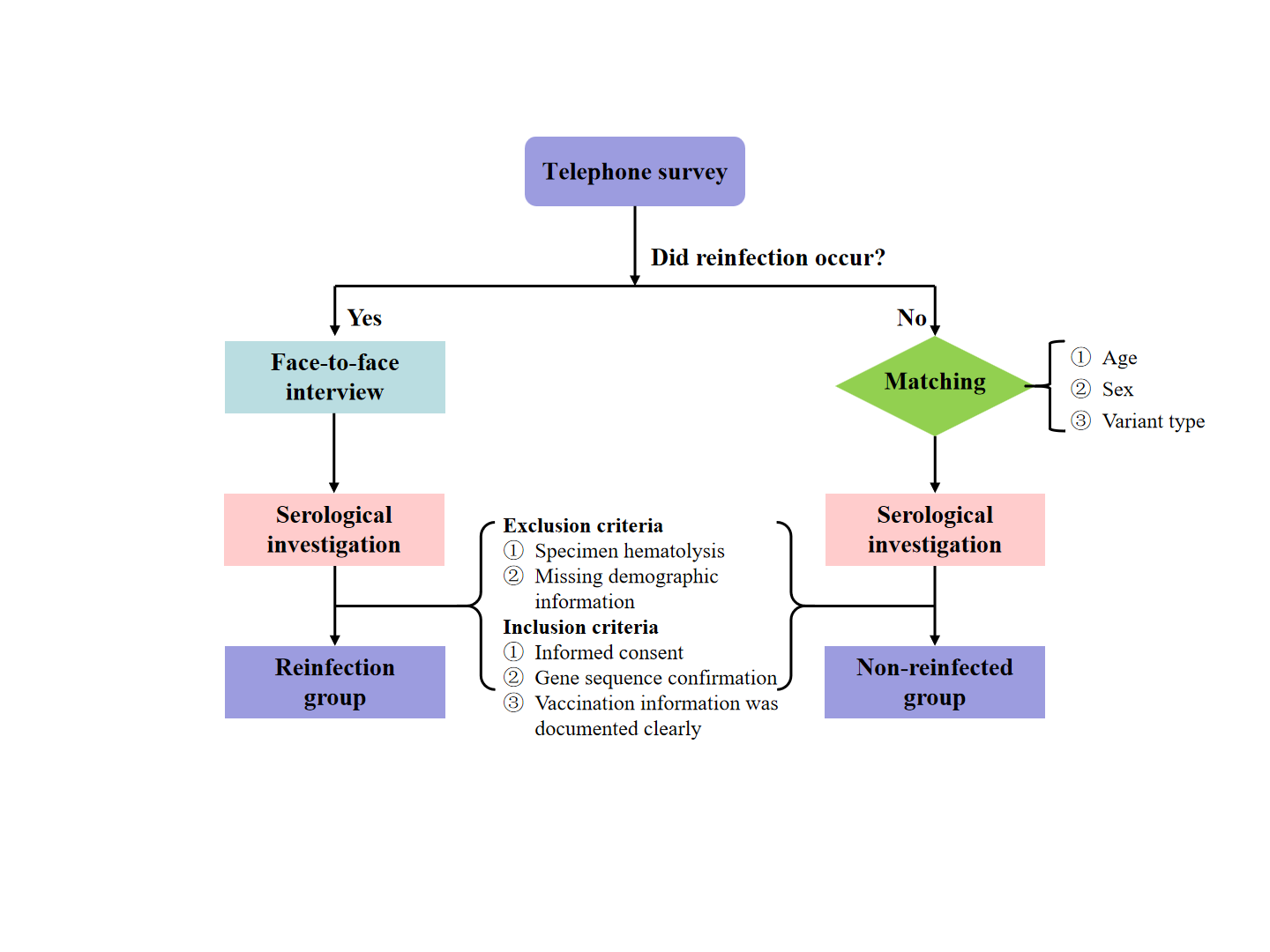

Supplement: Supplementary file 4 [file Image_1.TIF]
